# PIPDeploy: Development and implementation of a gamified table top simulation exercise to strengthen national pandemic vaccine preparedness and readiness

**DOI:** 10.1016/j.vaccine.2020.11.047

**Published:** 2021-01-08

**Authors:** Ioana Ghiga, Sol Richardson, Alba Maria Ropero Álvarez, Masaya Kato, Dhamari Naidoo, Satoko Otsu, Phuc Thi Nguyen, Phuong Nam Nguyen, Tim Nguyen

**Affiliations:** a Global Infectious Hazard Preparedness Department, WHO Health Emergencies Programme, World Health Organization, Geneva, Switzerland; b Institute of Psychiatry, Psychology & Neuroscience, King's College London, 16 De Crespigny Park, London SE5 8AF, United Kingdom; c World Health Organization, Regional Office for the Americas, Washington, United States; d World Health Organization, Regional Office for the Western Pacific, Manila, Philippines; e World Health Organization, Country Office in Nigeria, Abuja, Nigeria; f World Health Organization, Country Office in Viet Nam, Hanoi, Viet Nam

**Keywords:** Influenza, Vaccine response, Pandemic preparedness, Gamification, Simulation exercise, χ^2^, Chi square, APSED, Asia Pacific Strategy for Emerging Diseases and Public Health Emergencies, df, degrees of freedom, FEMA, United States Federal Emergency Management Agency, IHR, International Health Regulations, NDVP, National deployment and vaccination plans, PIP Framework, Pandemic Influenza Preparedness Framework, WHO, World Health Organization

## Abstract

Successful emergency vaccination campaigns rely on effective deployment and vaccination plans. This applies to localised outbreaks as well as for pandemics. In the wake of the 2009 H1N1 influenza pandemic, analysis of the global Vaccine Deployment Initiative, through which the World Health Organization (WHO) donated pandemic influenza vaccines to countries in need, revealed that an absence of vaccine deployment plans in many countries significantly hindered vaccine deployment. Through the Pandemic Influenza Preparedness Framework adopted by the World Health Assembly in 2011, WHO is engaging in several capacity building activities to improve pandemic influenza preparedness and response and make provisions for access to vaccines and sharing of other benefits. The Framework calls for the development and exercise of operational plans for deployment of influenza vaccines to enhance pandemic preparedness. To this end, WHO has supported the development of PIPDeploy, an interactive, in-person table top simulation exercise to facilitate learning for emergency preparedness. It employs various game design elements including a game board, time pressure, leaderboards and teams to enhance participants’ motivation. PIPDeploy formed part of five WHO Pandemic Influenza Vaccine Deployment Workshops attended by national-level managers responsible for pandemic influenza vaccine response predominantly in non-producing countries. The purpose of this study was to describe the features and application of PIPDeploy, and present findings of the evaluation of participants’ experiences during the simulation involving a “hot wash” discussion and collection of quantitative data. The simulation’s instructional approach was widely accepted by participants, who reported that the format was novel and engaging. They reflected on its utility for identifying gaps in their own vaccine deployment plans and regulatory frameworks for importation of vaccine products. All participants found the simulation relevant to their professional objectives. A range of other potential applications were suggested, including PIPDeploy’s adaptation to sub-national contexts and to other epidemic diseases.

## 1 Introduction

An influenza pandemic occurs when a novel influenza virus emerges and spreads rapidly in populations without natural immunity across different geographic regions. A severe pandemic can result in millions of deaths globally, with widespread social and economic effects. While annual global expenditure on pandemic preparedness (as of 2019) is estimated at US$ 4.5 billion per year [1], or less than US$ 1 per capita, this represents less than 1% the estimated global economic costs of a moderate to severe pandemic of around US$ 500 billion [2]. The global Coronavirus disease (COVID-19) pandemic and its health and economic impacts underscore the continuing importance of pandemic preparedness.

Vaccines represent a key tool for controlling influenza pandemics, while planning for vaccine deployment is key ensuring effective pandemic response. During pandemic emergencies, however, supply of appropriate vaccine is likely to be constrained by limited production capacity alongside the need to maintain output for seasonal influenza, and the fact that it is impossible to stockpile vaccines specific to novel viruses. These challenges for pandemic response are compounded for countries without local vaccine production or unable to acquire sufficient quantities of vaccines through their own means. Through the Pandemic Influenza Preparedness (PIP) Framework [3], adopted by the World Health Assembly in 2011 [4], WHO and partners are working to improve pandemic influenza preparedness and response capacities, establish fair, transparent and effective processes for sharing of influenza viruses with human pandemic potential, and strengthen countries’ protection against pandemic influenza. As a result of these efforts, it is expected that approximately 420 million doses of pandemic vaccines, 10 million treatment courses of antivirals and 250,000 diagnostic kits will be available to countries in most need in case of a pandemic influenza emergency [5].

### 1.1 Lessons learned since the 2009 H1N1 pandemic and ongoing challenges for pandemic preparedness

The 2009 H1N1 influenza pandemic resulted in an estimated 284,000 deaths worldwide in its first 12 months, and posed significant challenges for global pandemic vaccine response in terms of regulatory processes for assessing their immunogenicity and safety, estimating short- and long-term production capacity of manufacturers, and allocation of stocks among affected countries [6].

In response, WHO established the Pandemic Influenza A (H1N1) Vaccine Deployment Initiative, which brought together technical agencies, donor governments and private-sector organizations to deliver over 78 million doses of pandemic H1N1 vaccine to 77 countries from June 2009 to October 2010. Subsequent reviews of the pandemic response identified challenges pertaining to outdated and inflexible pandemic preparedness plans; poor timeliness of vaccine delivery to populations in need; gaps in public health communication strategies; and inadequate logistics planning and cold chain capacity. These highlighted the need for further development of operational plans for deployment of influenza vaccines by individual countries and regular engagement between WHO and major global stakeholders in readiness training for future pandemics, including simulation exercises. They also encouraged advance agreements between country authorities, vaccine manufacturers and other relevant parties to facilitate approval and delivery of pandemic vaccines to low-resource countries, increase equity in supply, and support advance planning for vaccine administration [7], [8]. An example of such an initiative to achieve these ends is the PAHO Revolving Fund mechanism, which has played a key role negotiating vaccines on behalf of countries and territories in the Americas, and was instrumental in securing early access to vaccines for 24 countries in Latin America during the 2009 influenza pandemic [9].

### 1.2 The importance of national deployment and vaccination plans

It has been noted that non-vaccine-producing countries or countries with no seasonal influenza vaccination face unique challenges for pandemic response including limited access to vaccines, delays in licensing and market authorization, and challenges in delivering to, and monitoring their use in, priority populations. The existence of seasonal influenza programmes may contribute to the development of national deployment and vaccination plans (NDVP), as these allow countries to test crucial regulatory and delivery systems, strengthen their national response to influenza, and prepare for future pandemic vaccine deployment. Through seasonal vaccination programs, countries are not only able to reduce the disease burden due to seasonal viruses, but also are learning how to reach high-risk groups not traditionally targeted in the routine immunization programmes, such as elderly, people with chronic diseases and pregnant women [10]. An analysis of the 97 countries eligible to receive WHO-donated vaccines as part of the Vaccine Deployment Initiative during the 2009 epidemic, of which >80% were classified as low-income or lower-middle income, found that those with a seasonal influenza vaccine program were more likely to submit a NDVP in a timely manner to their request for vaccine donation, and more ready to receive and use donated vaccines than those without [11]. In the absence of such experience at the national level, other approaches may be needed to accelerate development of NDVPs.

NDVPs could trigger establishment of processes and structures required for delivering pandemic vaccine and related supplies which may not exist in some non-producing countries or may be outdated [12]. At the global level, the most recent global survey conducted in 2018 found that only 60 out of 104 (58%) countries surveyed have developed some form of a NDVP. Only 24 mentioned, however, that they have a standalone NDVP, while 36 mentioned some provisions are included in wider influenza pandemic preparedness plans. Of the 44 which have not developed a NDVP, 35 intend to do so [13].

It is important to also consider the NDVPs in the wider context of health emergency preparedness as they directly contribute to capacity building efforts under the International Health Regulations (IHR) and regional strategic frameworks aimed to advance their implementation such as Asia Pacific Strategy for Emerging Diseases and Public Health Emergencies (APSED) [14].

### 1.3 Simulations for pandemic preparedness

Building on lessons from the 2009 H1N1 influenza pandemic, the PIP Framework calls for cooperation between the WHO and individual countries to develop NDVPs and plans for their implementation [3].

According to the WHO simulation exercise manual [15], “simulation exercises can help develop, assess, and test the functional capabilities of emergency systems”, and as a method for dissemination of knowledge on procedures and mechanisms to respond to outbreaks and public health emergencies. The manual identifies four types of simulation exercises to identify and address issues before an actual emergency occurs: table-top exercises, drills, functional exercises and field exercises. It is recommended that all types of exercise should be employed to form part of a comprehensive programme made up of elements with increasing complexity, with each exercise building on the previous one until they are as close to reality as possible.

These exercises should feed into the preparedness cycle, which comprises the stages of planning, organizing, training, equipping, exercising, evaluating, and taking corrective action [16].Table-top exercises typically take the form of a facilitated discussion around a scenario or narrative which promotes brainstorming and group problem solving, and, of the four exercise types, provide an informal, low-stress environment for facilitated discussion of an emergency situation, have lowest level of preparation, and are most suitable for development or review of response plans and familiarizing participants with their roles and responsibilities.

One activity under the PIP Framework has been the development of a table-top training and simulation exercise named PIPDeploy, which is designed to aid in identifying and correcting bottlenecks and failure points in vaccine delivery in public health emergencies [17], while incorporating game design elements to enhance user engagement.

### 1.4 Gamification and serious games

Gamification and serious games are related concepts, and their applications often share the same objectives. Although both have their origin in the 1980s, they have garnered increasing interest in recent years [18].

Serious games are fully-developed games serving a specific, non-entertainment purpose [19], [20]. They have been employed in a number of contexts, including in education [21], humanitarian settings [22], and training of health professionals [23] in which some trials have associated their use with improved patient outcomes [24].

Gamification, meanwhile, refers to the use of distinct game elements embedded in real-world, non-game contexts, which often reflect the rules-based, goal-oriented nature of games [19], [25] and have the potential to make non-game products and services more enjoyable and engaging [26]. Other definitions of gamification include “the process of using game thinking and game mechanics to solve problems and engage users” [27], or employing game elements to motivate specific behaviours [25].

The boundary between “game” and gamified application can be subjective and context-dependent; often this can only be determined from designers’ intentions or user experiences [19]. One definition of a game is that of “a system in which players engage in an artificial conflict, defined by rules, that results in a quantifiable outcome” [28]. Gamification, meanwhile is characterized by use of game “elements” [25]. It should be noted that addition of one informal rule or shared goal by a group of users may turn a “gamified” application into a “full” game, which is a composite of multiple necessary conditions [19].

### 1.5 Game design elements

In gamified contexts, game design elements, also referred to as “motivational affordances” [29] or “ludic elements” [30] create immersive experiences, interactions among participants, and motivation to achieve objectives [19], [21]. Various authors have proposed lists of game design elements [25], [31], which commonly include points, badges, leaderboards, performance graphs, meaningful stories, avatars, teammates, three-dimensional environments, competition, and time pressure.

### 1.6 Gamification and motivation

Carroll [32] has proposed that games motivate individuals through permitting and encouraging risk taking, engagement in “metaphoric cover stories”, and exploitation of curiosity by prompting participants to pose questions to themselves and later formulate answers to these questions [25], [33].

Various studies on gamification have made reference to self-determination theory, which proposes that individuals’ behaviour is driven by three psychological needs: the need for competence, the need for autonomy, and the need for social relatedness [34]. Competence refers to feelings of efficiency and success while interacting with the environment [35]. Autonomy refers to psychological freedom and volition to fulfil a given task [36]. Social relatedness, meanwhile, refers to feelings of belonging, attachment, and coherent integration within a social environment [37]. However, the degree to which gamification enhances motivation through psychological need satisfaction may depend on aspects such as their aesthetics, quality of execution, individual participants and their motivations [25].

### 1.7 Applications of gamification

Gamification has been applied in a range of contexts including work, finance, health and education [19], [25], [38]. Game design elements have increasingly been integrated into status and reward systems (e.g. airline miles) and in social media (e.g. virtual badges). Gamification has also been employed in public health emergency response training in the context of local public health departments [39].

A literature review of 128 empirical research papers on applications of gamification, with elements signalling achievement and progression being most common, found that most applications of game design elements were positively associated with learning outcomes [40].Meanwhile, a meta-analysis of 38 studies found that inclusion of game fiction and social interaction were significant moderators of the effect of gamification on behavioural learning outcomes [25]. Although the majority of results of individual applications of gamification have been positively-oriented, there is a wide variation in the outcome measures and quality of experimental design [40], [41]. One experimental study of effects of game design elements employed in different configurations in a digital simulation setting found that badges, leaderboards, and performance graphs were positively associated with need for competence [25]. Use of avatars, meaningful stories, and teammates, meanwhile, was associated with social relatedness.

### 1.8 Purpose statement

The purpose of this paper is to describe, and discuss the utility of, the application of a gamified simulation exercise developed with the support of the WHO aimed at strengthening national pandemic vaccine preparedness and readiness, and to present findings from qualitative and quantitative evaluations of the simulation exercise by participants.

## 2 Methods

PIPDeploy is a gamified in-person table-top simulation exercise developed in partnership with the non-profit consultancy firm GaneshAID [42] to facilitate learning for emergency preparedness among national-level managers responsible for pandemic influenza vaccine response such as national and regional officials responsible for immunization campaigns, regulatory authority representatives, chiefs of logistics and incident commanders. Its objectives are to: 1) provoke discussions on key gaps in preparing for or updating national pandemic influenza deployment plans; 2) enable conversations on best practices on in-country pandemic influenza vaccine deployment governance and operations; and 3) better understand country training needs and barriers to future participation in simulation exercises.

Its two primary features include a scenario based on a fictitious non-vaccine-producing country named Timoa in which participants move progressively through five themed missions, and purposive integration of a board-game-like mechanic in which participants collect cards representing resources for pandemic response.

PIPDeploy’s development was informed by an established instructional design model involving five phases [43], [44]. The analysis phase involved engagement between GaneshAid and WHO subject matter experts to determine its general content and to articulate learning objectives while account for learners’ context and their characteristics. During the design and development phases, discussions took place on how to engage users by creating a stimulating and interactive experience, and how to integrate training and simulation elements. In the implementation and evaluation phases, the prototype exercise was piloted to evaluate its usability, and refined based on feedback from participants. Summative evaluations were performed on the exercise’s finalized version over subsequent sessions.

Based on WHO’s 2012 guidance document [12], aspects of pandemic preparedness covered by the simulation included vaccination strategy and assessment of NDVPs, management of vaccine deployment operations, legal and regulatory planning, risk communication, logistics, including supply chain and waste management, post-deployment surveillance, termination of operations, and management of adverse effects following immunization.

### 2.1 Summary of the PIPDeploy simulation exercise

The simulation is led by an external facilitator and is divided into five sequential missions centred on a specific goal ranging from 55 to 85 min in length which take place over one day (Table 1) [45]. Participants are divided into teams of up to five, and either allocated to teams by country or distributed to achieve a mixture in terms of their real-world roles depending on the composition of workshop participants. Each mission is introduced with an animated video. The facilitator presents a specific challenge on which participants are asked to provide input and distributes written materials created for the exercise and other related documents which provide background information on the scenario and key concepts.

**Table 1:** Summary of PIPDeploy missions and objectives [45].

Mission	Title	Scenario	Goal	Objectives
Mission 1	National deployment and vaccination plan (NDVP)	A novel viral outbreak occurs in neighbouring countries	To revise or develop a NDVP to prepare for a potential influenza pandemic, and review relevant indicators for its assessment	• Objective 1: To define what constitutes a deployment and vaccination plan • Objective 2: To determine the indicators to be assessed in a NDVP for pandemic influenza • Objective 3: To explain the importance of assessing the NDVP • Objective 4: To define the structure of the chain of command and identify the roles and responsibilities of the three key actors involved in implementing an NDVP (Incident Commander, Chief of Logistics, and Chief of Vaccination) • Objective 5: To list key aspects of the welfare of human resources in pandemic situations and key actions to ensure that available staff are trained and capable of supporting pandemic vaccine deployment and vaccination operations
Mission 2	Legal and regulatory planning	Declaration of an influenza pandemic by the WHO	To address legal and regulatory planning and mechanisms for access to therapeutic pandemic products	• Objective 1: To describe the existing legal and regulatory requirements, both international and national, before an event and associated preparedness activities required to meet these requirements • Objective 2: To identify processes to ensure relevant authorities comply with all legal and regulatory requirements and complete the related procedures for importing, warehousing, packaging, shipping and using a vaccine before it is needed • Objective 3: To specify procedures that need to be followed to request a vaccine from the WHO
Mission 3	Public communication in a pandemic situation	Panic is spreading	To reflect on risk communication systems and communication challenges associated with vaccine deployment during an influenza pandemic	• Objective 1: To determine which evidence should be integrated into risk communication messaging to focus on changing or maintaining public behaviour in support of vaccination and trust in authorities • Objective 2: To list basic principles of communicating with the general public during an outbreak and identify challenges in convincing target groups to seek vaccinations • Objective 3: To define characteristics of evidence-based communications strategy, and plan for each stage of vaccine deployment (i.e. before, during, and after)
Mission 4	Communication, supply chain and waste management	Supply chain capacity is insufficient	To reflect on strategies and processes for ensuring timely deployment of vaccines and ancillary items and to plan for surge capacity	• Objective 1: To identify the supply chain management processes required to successfully deploy vaccines and ancillary items within seven days • Objective 2: To document capacity a supply chain logistic system, identify gaps, and prepare surge capacity along with a budget • Objective 3: To collect information to manage vaccine deployment and update national pandemic preparedness plans • Objective 4: To plan and manage the safe disposal of used injection equipment, vaccine vials and other hazardous medical waste, and ensure that waste management systems can deal with the additional waste generated by a pandemic response • Objective 5: To record the management process for handling waste in a management information system
Mission 5	Post-deployment surveillance system and management of adverse effects following immunization (AEFI)	Monitoring performance and safety of a novel pandemic influenza vaccine	To assess the post-deployment surveillance system and management of AEFIs	• Objective 1: To discuss how enhanced post-deployment surveillance can be included in a NDVP • Objective 2: To describe how to evaluate post-deployment surveillance operations and ensure their correct implementation during an influenza pandemic • Objective 3: To describe managerial functions for planning, investigating, responding to and monitoring AEFIs • Objective 4: To identify activities that should take place on termination of vaccine deployment to enable (among other things) identification of sections of the NDVP to be updated

Participants spend the majority of time in each mission brainstorming possible solutions to the challenges presented in each mission, presenting their findings to other teams, and then comparing solutions between teams and providing feedback.

**Fig. 1:**
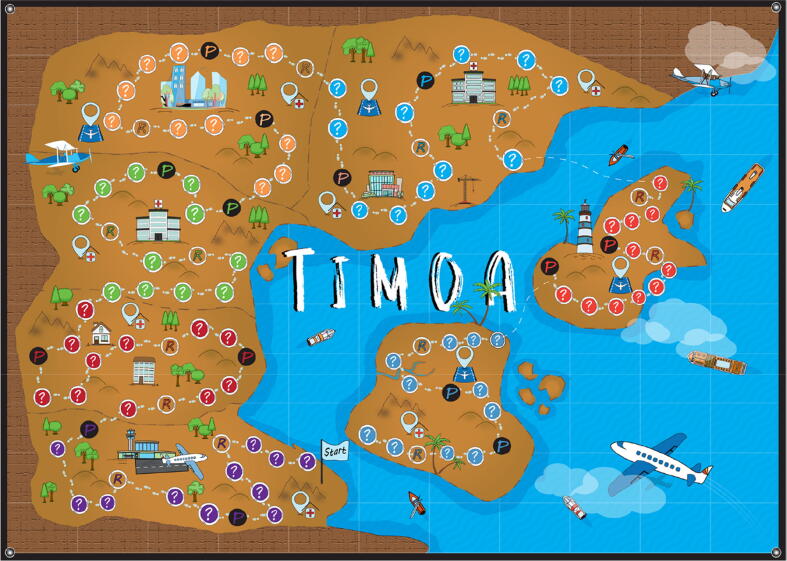
Illustration of the PIPDeploy game board. Teams of participants move across the game board by rolling dice. A resource card is drawn when participants land on a resource circle (R). A penalty card is drawn when participants land on a penalty circle (P). The mission ends once 10 min have elapsed or the final circle on the game board is reached [47].

The last 10 min of each mission involve collecting “resource cards” of different types by rolling dice to move across the game board and either landing on a “resource circle” or correctly answering questions when they land on a “resource circle” (Fig. 1). Resource cards also include a “gold card” which can represent any resource. Resources represent activities that must be completed for increasing country-level preparedness, and are specific to each mission. The board also includes “penalty circles”, representing preparedness activities that were not completed, and a “penalty card” is drawn which nullifies a corresponding resource card. Cards are placed on resource boards, which display a grid representing squares with squares representing different types of resources corresponding to the cards, with the objective of completing horizontal, vertical or diagonal lines. The team which completes the most lines is considered the “winner”.

At the end of each mission, participants are given “mission rewards”, consisting of key take-home messages and infographics. This is followed by a “hot-wash^1^” discussion led by the facilitator [46], to synthesize of key concepts and knowledge of planning for deployment of vaccines for pandemic response. Sessions are observed by an evaluator whose role is to identify key areas where further training may be needed and help refine learning objectives of the game in future iterations.

Finally, the day ends with a 30-min hot wash session, in which participants review the resources gathered and discuss their value in achieving a state of readiness, and synthesize the lessons learned over the five missions to reflect on their own country’s situation and identify current strengths and weaknesses in preparedness for deployment of pandemic vaccines.

### 2.2 Piloting PIPDeploy

PIPDeploy was piloted over two full-scale pilot sessions, whose objectives were to 1) evaluate participants’ acceptance of the exercise’s learning approach; 2) evaluate the learning content, allotment of time, participants’ engagement and interactions; 3) gather feedback and suggestions for improvement through discussion with participants and an evaluation questionnaire; and 4) inform revisions for subsequent versions. While the format of PIPDeploy remained similar between the two pilots, two notable changes were made. First, the terminology of some of the game elements, the number of question cards and their phrasing were modified. Second, while the exercise was initially intended to promote collaboration and did not include the paradigm of a winner, an element of competition was added through the addition of resource boards which were clearly visible to all teams and allowed for comparison of achievements. Although a “winning” team was identified, teams were encouraged to collaborate through exchange of resources [45], [47]. In its final form, PIPDeploy included the common game design elements of points, leaderboards, performance graphs, meaningful stories, avatars, teammates, three-dimensional environments, competition, and time pressure.

### 2.3 PIPDeploy simulations

To date, PIPDeploy exercises have taken place at WHO Pandemic Influenza Vaccine Deployment Workshops in Washington, D.C., United States,^2^ from 5 to 7 February 2019; Dushanbe, Tajikistan,^3^ from 30 April–2 May 2019; Manila, Philippines,^4^ from 17 to 19 September 2019; Lagos, Nigeria,^5^ from 4 to 6 November 2019 and Hanoi, Viet Nam, from 11 to 12 December 2019 [5], [48], [49], [50], [51]. The first two of these simulation exercises were part of PIPDeploy’s pilot phase [45], [47].

The workshops usually took place over three days. Although content varied between workshops, their overall purpose was to 1) facilitate updates to, or development of NDVPs, 2) strengthen collaboration between different sectors and stakeholders involved in pandemic influenza deployment operations, 3) strengthen regulatory systems to ensure timely allocation of pandemic supplies, and 4) conduct the PIPDeploy simulation exercise to enhance preparedness and test pandemic response plans [49]. The workshops emphasised the strategic importance of pandemic influenza vaccines as an integral part of pandemic preparedness, and the need to identifying key gaps and weaknesses in NDVPs. Depending on countries’ progress in developing comprehensive NDVPs, participants were also asked to complete the draft WHO Influenza Vaccine Request Form [48], [49] and to share their feedback and suggestions for the form’s improvement with workshop organizers.

### 2.4 Workshop evaluation

Participants were invited to qualitatively evaluate the PIPDeploy simulation exercise at the end of each workshop to provide their opinions and feedback to identify further improvements and determine whether the sessions’ objectives had been achieved. We identified common themes across participants’ responses during the hot was discussion. A questionnaire was also administered to provide quantitative data following three workshops [5], [48], [49]. In addition to a binary choice question on whether the simulation met participants’ individual objectives and was relevant to their job, participants responded to 12 other question items on a four-point Likert scale. We performed a descriptive analysis of participants’ responses to these questions and performed chi square (χ^2^) tests to investigate participants’ scores to each question varied between each of the three workshops (please see the online supplementary material) using Stata 16.

## 3 Results

Overall, the qualitative session evaluations found that participants had a clear understanding of the rules and purpose of the PIPDeploy simulation, and considered it an engaging tool for imparting knowledge on deployment and vaccination planning [48]. Its instructional approach, based on engagement in a simulated problem and activation of existing knowledge through the scenarios presented, demonstration of new knowledge through facilitator presentations and engagement with background materials, and application and integration of new knowledge through brainstorming and presenting solutions and competing for resources [44], was widely accepted by participants. In general, its structure, which categorized steps involved in national deployment and vaccination plans in a systematic and linear design with progression through sequential missions, was considered one of its key advantages. This aspect was especially useful for participants from countries where NDVPs were less developed, and presented an opportunity for them to identify gaps in their own systems and consider how to tackle them [51].

Participants were attracted by the novelty of the format and interactive experience [48]. They also praised the simulation’s delivery, both in terms of the facilitators and design of the game board and other materials. PIPDeploy allowed for experimentation and learning in a “stress-free” simulated environment, and participants commented that this aspect contributed to their productivity during the session. At the same time, time-pressure contributed to intensive problem-solving and collaboration within teams [5].

The example of a fictitious country and scenario, which was at the same time specific but sufficiently generalizable and relevant to participants’ experiences at the national level, encouraged them to reflect on the capacity of their own national systems, apply their knowledge in a practical setting, and think concretely about potential real-life situations [48], [49], [51]. The scenarios presented, and teams composed of experts and leaders whose experience encompassed multiple aspects of pandemic vaccine response, stimulated teamwork, critical thinking and knowledge exchange [48]. The encouragement of teamwork highlighted the benefits of strong collaboration between national-level agencies and sectors for ensuring effective pandemic response [5]. Participants were given the opportunity to understand the differences and commonalities between their roles and responsibilities and those of their teammates, the flow of responsibility from the central level to the district and community levels, and how they could effectively coordinate their activities in a pandemic scenario [48], [51]. This was particularly the case during the session in held in Nigeria, which was attended by representatives from four federal government agencies and national influenza sentinel sites [5].

While in its first pilot iteration participants were anticipating competition and were attempting to compare their progress with those of other teams [47], [48], the injection of a competitive element in subsequent iterations was a welcome addition and gave further impetus to teams’ performance.

General suggestions included adapting PIPDeploy to other epidemic threats such as Ebola and Lassa fever [5] and for scenarios involving multiple-dose pandemic vaccines, and for subnational pandemic response scenarios [48]. Several respondents expressed their interest in applying PIPDelploy at the national level during pandemic readiness exercises, but with adaptations to emphasize identification of risks and challenges specific to the country setting.

Possible areas for improvement included translating PIPDeploy to other languages to allow participants to use their native language [47], [49], making questions shorter or more specific, giving greater context to the scenarios, increasing the time for discussion during the brainstorming sessions at the beginning of each mission, including public communication experts in the simulation to allow them to share their perspectives. Participants also suggested that sessions involving participants from multiple WHO Regions could be run to allow further exchange of experiences and identify common lessons. Information and summaries from the qualitative evaluation of the session can be found in the relevant reports [5], [45], [47], [48], [49].

All 50 of the 69 respondents who provided data across the three workshops agreed with the statement that the simulation met their individual objectives and was relevant to their job [5], [48], [49]. In addition, 100% of respondents who provided data (n = 79) agreed that they “learned one or more things [would] will allow [them] to increase preparedness and readiness for deployment of pandemic influenza vaccine in [their] country”. There was a significant change in scores for certain items between simulations; for example, compared with those attending the pilot simulation in Washington, D.C., United States, participants who attended simulations held in Dushanbe, Tajikistan (χ^2^ = 19.3, degrees of freedom [df] = 2, p < 0.001), and Lagos, Nigeria (χ^2^ = 6.5, df = 2, p < 0.038), indicated stronger agreement with the statement “the game play was well organized and structured”. This suggests improvement of session delivery over time, which may reflect facilitators’ increased familiarity of the session format and common issues raised by participants. The full results of the quantitative evaluation are shown in the online supplementary material.

## 4 Discussion

PIPDeploy was positively appraised and widely accepted among participants. Game design elements contributed to their engagement in the simulation and learning outcomes. Introduction of a “winning” mechanic promoted engagement through competition between teams, the use of a game board to displayed linear progress through missions, and collection of resources demonstrated participants’ progress and competence in meeting mission objectives. Fulfilment of their need for autonomy was enhanced through opportunities to take risks to formulate novel solutions to challenges within a low-pressure simulated environment [30], [32], and relating these to their own experiences at the national level while testing current readiness planning. Participants also experienced social relatedness through cooperation within teams, and between teams at the end of the simulation.

The inter-pandemic period presents an opportunity to take actions to maximize the efficiency and effectiveness of vaccine response in subsequent pandemics [52]. PIPDeploy encourages participants to apply lessons learned and address shortcomings of current strategies for influenza vaccine response at the country level, and a basis on which to plan for more complex exercises with country-specific content as part of the preparedness cycle. The simulation integrated a wide range of game design elements to promote participants’ engagement; while each of the individual elements have been employed either individually or in combination, PIPDeploy represents (to our knowledge) the first attempt to purposively integrate the full range of game design elements previously described [25], [31] alongside a board-game like mechanic (itself an adaptation of a performance graph within a three-dimensional environment) in the context of a pandemic preparedness simulation exercise. The workshops stimulated collaboration between participants representing a range of sectors and stakeholders involved in pandemic influenza deployment and have been broadly successful in meeting their objectives; participants’ experiences have catalysed further work towards improving influenza pandemic preparedness at the national level as countries have established working groups for development of NDVPs, reviewed their regulatory systems for influenza vaccine products, bolstered training of relevant personnel on NDVPs, and tested NDVPs through further simulation exercises [50]. It is hoped that these efforts may expedite countries’ access to, and deployment of, essential vaccines, and allow them to limit the human and economic costs of potential future influenza pandemics.

The development of NDVPs and capacity building for influenza preparedness as a result of PIPDeploy may also provide a foundation for vaccine response in the face of other pandemic threats. For example, once a safe and effective vaccine for COVID-19 becomes a reality, individual countries face the need to develop specific NDVPs, including strategies for delivery of vaccine to priority populations and post vaccination surveillance, to support the unprecedented challenge of its mass global distribution [53].

## Funding

This study was funded by the World Health Organization.

Disclaimer

This is an Open Access article published under the CC BY 3.0 IGO license which permits unrestricted use, distribution, and reproduction in any medium, provided the original work is properly cited. In any use of this article, there should be no suggestion that WHO endorses any specific organisation, products or services. The use of the WHO logo is not permitted. This notice should be preserved along with the article’s original URL.

The authors alone are responsible for the views expressed in this article and they do not necessarily represent the views, decisions or policies of the institutions with which they are affiliated.

## Declaration of Competing Interest

The authors declare that they have no known competing financial interests or personal relationships that could have appeared to influence the work reported in this paper.

## Footnotes

1 According to the United States Federal Emergency Management Agency (FEMA), the definition of a “hot wash” is “A facilitated discussion held immediately following an exercise among exercise participants from each functional area that is designed to capture feedback about any issues, concerns, or proposed improvements players may have about the exercise.” It represents an opportunity for participants to voice their opinions on the exercise and their own performance. Evaluators, who should take notes, can also seek clarification on certain actions and what prompted participants to take them [46].

2 The PIPDeploy exercise was attended by participants representing the Dominican Republic, Columbia, Suriname, Haiti, El Salvador, Paraguay, Brazil, Mexico and Jamaica.

3 The PIPDeploy exercise was attended by participants representing Armenia, Azerbaijan, Georgia, Kazakhstan, Kyrgyzstan, Tajikistan, Turkmenistan, and Uzbekistan.

4 The PIPDeploy exercise was attended by participants representing Cambodia, China, the Lao People’s Democratic Republic, Mongolia, Philippines, and Viet Nam.

5 The PIPDeploy exercise was attended by participants Nigeria only, with participants representing the National Primary Health Care Development Agency (NPHCDA), National Agency for Food and Drug Administration and Control (NAFDAC), and Nigeria Center for Disease Control (NCDC).
