# Supplementary material for: PIPDeploy: Development and implementation of a gamified table top simulation exercise to strengthen national pandemic vaccine preparedness and readiness
Source: Vaccine. 2021 Jan 8;39(2):364–71. doi: 10.1016/j.vaccine.2020.11.047 (PMC7805265; doi:10.1016/j.vaccine.2020.11.047)
Supplement: Supplementary Data 1 [file mmc1.docx]

# Online supplement

## Methods

We conducted an analysis of quantitative data obtained from participants during evaluations following three PIPDeploy simulations: A (Washington, D.C., United States), B (Dushanbe, Tajikistan), and C (Lagos, Nigeria). Data were obtained from the relevant workshop reports [^[[1]](#endnote-1)^,^[[2]](#endnote-2)^,^[[3]](#endnote-3)^].

In addition to providing binary responses to the statement “the game play met my individual objectives and was relevant to my job” indicating either agreement or disagreement, participants were requested to give scores on a further 12 numbered statements on a four-point Likert scale (range: 1–4) to indicate their level of agreement.

We conducted a descriptive and statistical analysis of this quantitative data. First, for each simulation session we compiled the distributions of scores for each statement by level of agreement, and calculated the percentage of participants indicating agreement (i.e. corresponding to scores of “3” or “4”) and mean scores with standard errors. Next, we performed a statistical analysis using chi square tests to investigate whether the distributions of respondents’ scores for each of the 12 statements differed between the three PIPDeploy simulations (i.e. A vs. B, A vs. C, and B vs. C). We calculated p-values based on the distribution of χ^2^ with two degrees of freedom; p-values of ≤0.05 were considered to signify a statistically significant difference in scores between simulations.

## Results

All 50 of the 69 respondents who provided data across the three workshops agreed with the statement that the simulation met their individual objectives and was relevant to their job. The numbering of the 12 further statements, numbers of respondents for each session evaluation, and descriptive analysis of participant responses are shown in Table 1.

| **Table 1. Descriptive analysis of participant agreement scores for 12 statements, based on evaluations of three PIPDeploy simulation sessions [i,ii,iii]** | | | | | | | | | | | | | | | | | | |
| --- | --- | --- | --- | --- | --- | --- | --- | --- | --- | --- | --- | --- | --- | --- | --- | --- | --- | --- |
| **Statement** | **WHO Pandemic Influenza Vaccine Deployment Workshop, Washington, D.C., United States, 5–7 February 2019 (A) [i] (n of participants=30)** | | | | | | **WHO Pandemic Influenza Vaccine Deployment Workshop, Dushanbe, Tajikistan, 30 April–2 May 2019 (B) [ii] (n=29)** | | | | | | **WHO Pandemic Influenza Vaccine Deployment Workshop, Lagos, Nigeria, 4–6 November 2019 (C) [iii] (n=21)** | | | | | |
|  | **Score*** | | | | **% agree** | **Mean (SE)** | **Score** | | | | **% agree** | **Mean (SE)** | **Score** | | | | **% agree** | **Mean (SE)** |
|  | **1** | **2** | **3** | **4** |  |  | **1** | **2** | **3** | **4** |  |  | **1** | **2** | **3** | **4** |  |  |
| 1. The game play was well organized and structured | 0 | 2 | 19 | 9 | 93.3 | 3.2 (0.1) | 0 | 0 | 4 | 25 | 100.0 | 3.9 (0.1) | 0 | 0 | 7 | 13 | 100.0 | 3.7 (0.1) |
| 2.The instructions were clear and comprehensive | 0 | 7 | 20 | 3 | 76.7 | 2.9 (0.1) | 0 | 0 | 7 | 22 | 100.0 | 3.8 (0.1) | 0 | 0 | 7 | 13 | 100.0 | 3.7 (0.1) |
| 3. The exercise scenario was adequately designed for its use | 0 | 3 | 16 | 11 | 90.0 | 3.3 (0.1) | 0 | 0 | 4 | 25 | 100.0 | 3.9 (0.1) | 0 | 0 | 12 | 7 | 100.0 | 3.4 (0.1) |
| 4. Participants were encouraged to play an active role in the game | 0 | 0 | 12 | 18 | 100.0 | 3.6 (0.1) | 0 | 0 | 4 | 26 | 100.0 | 3.9 (0.1) | 0 | 1 | 5 | 14 | 95.0 | 3.7 (0.1) |
| 5. The videos and briefings helped me understand and become engaged in the scenario | 0 | 1 | 10 | 19 | 96.7 | 3.6 (0.1) | 0 | 0 | 5 | 24 | 100.0 | 3.8 (0.1) | 0 | 0 | 6 | 14 | 100.0 | 3.7 (0.1) |
| 6. The game documents (injects, scenarios, cards) are adequately designed for their use | 0 | 2 | 13 | 14 | 93.1 | 3.4 (0.1) | 0 | 0 | 4 | 25 | 100.0 | 3.9 (0.1) | 0 | 0 | 9 | 10 | 100.0 | 3.5 (0.1) |
| 7. Handouts were relevant | 0 | 1 | 10 | 17 | 96.4 | 3.6 (0.1) | 0 | 0 | 2 | 27 | 100.0 | 3.9 (0.0) | 0 | 0 | 7 | 13 | 100.0 | 3.7 (0.1) |
| 8. The exercise helped me to identify some of my strengths as well as some of the gaps in my understanding of response systems, plans and procedures | 0 | 1 | 8 | 21 | 96.7 | 3.7 (0.1) | 0 | 0 | 9 | 20 | 100.0 | 3.7 (0.1) | 0 | 0 | 10 | 10 | 100.0 | 3.5 (0.1) |
| 9. The level and mix of disciplines and participants included the right people for this game play | 0 | 1 | 20 | 9 | 96.7 | 3.3 (0.1) | 0 | 0 | 5 | 24 | 100.0 | 3.8 (0.1) | 0 | 0 | 9 | 12 | 100.0 | 3.6 (0.1) |
| 10. The exercise improved my understanding of my role and function during an emergency response | 0 | 1 | 11 | 18 | 96.7 | 3.6 (0.1) | 0 | 0 | 6 | 23 | 100.0 | 3.8 (0.1) | 0 | 0 | 6 | 14 | 100.0 | 3.7 (0.1) |
| 11. I learned one or more things which will allow me to increase preparedness and readiness for deployment of pandemic influenza vaccine in my country | 0 | 0 | 10 | 20 | 100.0 | 3.7 (0.1) | 0 | 0 | 7 | 22 | 100.0 | 3.8 (0.1) | 0 | 0 | 5 | 15 | 100.0 | 3.8 (0.1) |
| 12. I would recommend playing this game to my colleagues | 0 | 1 | 4 | 25 | 96.7 | 3.8 (0.1) | 0 | 0 | 2 | 27 | 100.0 | 3.9 (0.0) | 0 | 0 | 4 | 17 | 100.0 | 3.8 (0.1) |

1: strongly disagree; 2: disagree; 3: agree; 4: strongly disagree; SE: standard error. Agreement with each statement was defined as a score of 3 or 4. SE: standard error.

The percentage of respondents who participated in the PIPDeploy simulation held in Washington, D.C., United States (A) in agreement with each of the 12 statements ranged from 76.7% (statement 2) to 100.0% (statements 4 and 11). All respondents participating in simulations held in Dushanbe, Tajikistan, and Lagos, Nigeria (B and C) indicated agreement with each of the 12 statements except for one respondent in simulation C who did not agree with statement 4 (“participants were encouraged to play an active role in the game”).

Chi square values and p-values for differences in distribution of respondent scores in response to each statement are shown in Table 2. Test results indicating a significant difference in scores between sessions are highlighted. The results show that there were significant differences in the distribution of scores between sessions for individual statements. Participants who attended workshop B (Dushanbe, Tajikistan) gave significantly different scores for statements 1, 2, 3, 4, 6 and 7 (in each case with responses indicating stronger agreement) compared with those who attended workshop A (Washington, D.C., United States). Meanwhile, participants who attended simulation C (Lagos, Nigeria) gave significantly different scores for statements 1 and 2 (with stronger agreement). Participants at workshop C gave lower scores than those at workshop B to some statements, with scores for statements 6, 7 and 9 having significantly different distributions between sessions.

| **Table 2. Statistical analysis of differences in distributions of participant agreement scores for 12 statements following three PIPDeploy simulation sessions: A (Washington, D.C., United States, 5–7 February 2019), B (Dushanbe, Tajikistan, 30 April–2 May 2019), and C (Lagos, Nigeria, 4–6 November 2019)** | | | | | | |
| --- | --- | --- | --- | --- | --- | --- |
| **Statement** | **A vs. B** | | **A vs. C** | | **B vs. C** | |
|  | **χ^2^ (df=2)** | **p** | **χ^2^ (df=2)** | **p** | **χ^2^ (df=2)** | **p** |
|  |  |  |  |  |  |  |
| 1. The game play was well organized and structured | **19.3** | **<0.001** | **6.5** | **0.038** | 3.1 | 0.080 |
| 2.The instructions were clear and comprehensive | **27.7** | **<0.001** | **18.2** | **<0.001** | 0.7 | 0.408 |
| 3. The exercise scenario was adequately designed for its use | **16.6** | **<0.001** | 2.1 | 0.351 | **12.6** | **<0.001** |
| 4. Participants were encouraged to play an active role in the game | **5.5** | **0.020** | 2.5 | 0.298 | 2.8 | 0.244 |
| 5. The videos and briefings helped me understand and become engaged in the scenario | 3.2 | 0.199 | 0.8 | 0.674 | 1.1 | 0.293 |
| 6. The game documents (injects, scenarios, cards) are adequately designed for their use | **9.9** | **0.007** | 1.4 | 0.504 | **6.6** | **0.010** |
| 7. Handouts were relevant | **8.6** | **0.014** | 0.8 | 0.687 | **6.2** | **0.013** |
| 8. The exercise helped me to identify some of my strengths as well as some of the gaps in my understanding of response systems, plans and procedures | 1.1 | 0.587 | 3.3 | 0.196 | 1.8 | 0.181 |
| 9. The level and mix of disciplines and participants included the right people for this game play | **16.8** | **<0.001** | 4.1 | 0.126 | **4.0** | **0.046** |
| 10. The exercise improved my understanding of my role and function during an emergency response | 3.1 | 0.216 | 1.0 | 0.603 | 0.6 | 0.456 |
| 11. I learned one or more things which will allow me to increase preparedness and readiness for deployment of pandemic influenza vaccine in my country | 0.6 | 0.436 | 0.4 | 0.529 | 0.0 | 0.945 |
| 12. I would recommend playing this game to my colleagues | 1.7 | 0.422 | 1.0 | 0.617 | 1.7 | 0.192 |

Statistically significant chi square test results for differences in distributions of participant

agreement scores between sessions are highlighted.

χ2: chi square value; df: degrees of freedom.

## Discussion

The results of the descriptive analysis (Table 1) indicate participants’ strong agreement with all 12 statements across the three simulation sessions, as evidenced by the high mean agreement scores and percentages of respondents in agreement with each statement. In sum, these results can be interpreted as showing that participantes expressed a very high degree of satisfaction with the PIPDeploy simulation, in terms not only of the content and organization of the sessions, but also in terms of their engagement and learning. Importantly, and consistent with PIPDeploy’s purpose of promoting learning for pandemic preparedness, all participants across the three simulations who provided data (n=79) indicated that they had they “learned one or more things that [would] allow [them] to increase preparedness and readiness for deployment of pandemic influenza vaccine in [their] country” (statement 11).

The results of the statistical analysis suggest that participants’ appraisals of session organisation, clarity of instructions, design of the scenarios, and suitability and relevance of handout materials improved between simulation sessions, particularly in session B and C when compared with the pilot session (A) held in Washington, D.C., United States. This may be a result of increasing familiarity of organizers with the format of the sessions, improvement of delivery, or successful adaptations following lessons learned following the pilot session. In addition, it may be speculated that improvement in agreement scores for statement 4 (“participants were encouraged to play an active role in the game”) in session B compared with session A, may be related to the introduction of a “winning” mechanic into the simulation and a resulting increase in participant engagement.

However, the results of this analysis should be interpreted with caution. The causal mechanisms for differences in distributions of scores between simulations could not be determined; these changes may have been due to differences in the composition of participant teams (e.g. in terms of language, nationality, job role and length of professional experience). The sample sizes and available covariate data were insufficient to adjust for potential confounding factors.

## References

1. [] World Health Organization. Report of the Pandemic Influenza Vaccine Deployment Workshop, Washington, 05–07 February 2019. Geneva: World Health Organization; 2019. [↑](#endnote-ref-1)
2. [] World Health Organization. Report of the WHO Pandemic Vaccine Deployment Workshop. Dushanbe, Tajikistan, 30 April–2 May 2019. Geneva: World Health Organization; 2019. [↑](#endnote-ref-2)
3. [] World Health Organization. Report of the WHO Pandemic Vaccine Deployment Workshop Lagos, Nigeria, 04–06 November 2019. Geneva: World Health Organization; 2019 [↑](#endnote-ref-3)
